# Titanium and Zinc Based Nanomaterials in Agriculture: A Promising Approach to Deal with (A)biotic Stresses?

**DOI:** 10.3390/toxics10040172

**Published:** 2022-03-31

**Authors:** Sónia Silva, Maria Celeste Dias, Artur M. S. Silva

**Affiliations:** 1 Associated Laboratory for Green Chemistry of the Network of Chemistry and Technology, Department of Chemistry, Campus Universitário de Santiago, University of Aveiro, 3810-193 Aveiro, Portugal; artur.silva@ua.pt; 2 Centre for Functional Ecology, Department of Life Sciences, University of Coimbra, Calçada Martim de Freitas, 3000-456 Coimbra, Portugal; celeste.dias@uc.pt

**Keywords:** drought, metal stress, phytotoxicity, phytopathogens, salinity, stress mitigation

## Abstract

Abiotic stresses, such as those induced by climatic factors or contaminants, and biotic stresses prompted by phytopathogens and pests inflict tremendous losses in agriculture and are major threats to worldwide food security. In addition, climate changes will exacerbate these factors as well as their negative impact on crops. Drought, salinity, heavy metals, pesticides, and drugs are major environmental problems that need deep attention, and effective and sustainable strategies to mitigate their effects on the environment need to be developed. Besides, sustainable solutions for agrocontrol must be developed as alternatives to conventional agrochemicals. In this sense, nanotechnology offers promising solutions to mitigate environmental stress effects on plants, increasing plant tolerance to the stressor, for the remediation of environmental contaminants, and to protect plants against pathogens. In this review, nano-sized TiO_2_ (nTiO_2_) and ZnO (nZnO) are scrutinized, and their potential to ameliorate drought, salinity, and xenobiotics effects in plants are emphasized, in addition to their antimicrobial potential for plant disease management. Understanding the level of stress alleviation in plants by these nanomaterials (NM) and relating them with the application conditions/methods is imperative to define the most sustainable and effective approaches to be adopted. Although broad-spectrum reviews exist, this article provides focused information on nTiO_2_ and nZnO for improving our understanding of the ameliorative potential that these NM show, addressing the gaps in the literature.

## 1. Introduction

Nanotechnology is gaining prominence in the agro-food system as consequence of the positive reports released in the last half decade highlighting the promising applications that nanomaterials may have in plant fortification, enhancing crop tolerance to abiotic stresses, and improving plant defence against pathogens [1,2,3,4,5,6,7,8,9,10,11,12]. Besides, nanotechnology offers a route to make agriculture more sustainable and precise, as it can contribute to the reduction of the amount of agrochemicals used in farming and their consequent accumulation in the environment, decrease the production cost of conventional fertilizers, smartly deliver active molecules to enhance crop performance and improve plant disease prevention and control, and mitigate the effects of environmental pollutants, pesticide degradation, micronutrients for efficient use, etc. [13]. Thanks to the reduced size of nanomaterials (NM), they show an increased surface-to-volume ratio, reactivity, and frequently distinct properties from their bulk or ionic counterparts [14]. Their particular physicochemical properties enable them to increase the efficiency of agrochemicals at the same time as decreasing the number of active compounds and raw materials needed to produce them and to be used in agriculture. These features will decrease the environmental impact of agrochemicals and contribute to the development of new strategies to overcome the challenges of modern agriculture, including climate changes, crop diseases, water limitations, and contaminated/poor soils, which altogether result in crop yield decline and food insecurity [15].

The general impact of NM in plants has been reviewed recently [16,17,18,19,20], as well as the use of nanocarriers to deliver active compounds to plants [21,22,23], the broad applications of NM to improve plant growth and plant stress tolerance [24,25,26,27,28,29,30,31], and the potential of NM for plant disease management [32]. Here, we focus on TiO_2_ (nTiO_2_) and ZnO (nZnO) nano-formulations as plant protective and ameliorative agents against (a)biotic stress based on the reports released in the last five years of research, highlighting their potential to cope with drought and salinity stresses, as well as their potential in metal stress mitigation and plant protection. This approach will allow readers to gain comprehensive knowledge about the potential applications of these materials as stress mitigators, as nano-fertilizers, and antimicrobial solutions for agrocontrol, in seed science, in growth promotion, metal remediation, etc.

## 2. Metal-Based Nanomaterials and Their Impact on Plants

Collectively, NM hold great promise for boosting crop production, as well as offering a safer alternative to synthetic counterparts for the environment when adopting green-synthesis to produce them. Metal-based NM show desirable characteristics that can be further explored and used in multiple agricultural proposes: higher effectiveness against plant pathogens, either due to biocide traits or to the modulation of plant defense mechanisms and of the proteome/metabolome [33,34,35]; stimulatory effects, improving plant physiological attributes and regulating plant metabolism [3,4,36]; prophylactic activity against adverse climatic/environmental conditions by promoting nutrient uptake/accumulation, modulating the antioxidant response, etc. [5,6,37]; smart delivery to plants of fertilizers, biostimulants, and pesticides as a result of controlled release of bioactive compounds [7]; and binding phases for both organic and inorganic contaminants, making them promising strategies for the remediation of polluted soils/waters [8,9,10,38].

After entering the plants by leaves and roots, nTiO_2_ and nZnO interact with plants at different levels, leading to several molecular, biochemical, and physiological changes [11]. The effects, positive or negative, depend on several factors, such as nanoparticles’ chemical nature, reactivity, size, and concentration [12]. Despite the large amount of information available on the effects of NM on the expression level of many genes and cellular mechanisms, much more needs to be done to unravel the mechanisms of action triggered by these nanoparticles (NP) [12].

One of the main processes improved by nTiO_2_ and nZnO is photosynthesis. In the case of the nTiO_2_, this may result from the photocatalytic properties of these NP, increasing the water hydrolyzation induced by light into oxygen, electrons, and protons. The electrons and protons produced enter the electron transport chain during the light reaction phase of photosynthesis, leading to a general increment of the photosynthetic process [39]. Besides that, biochemical and molecular studies also pinpoint an enhancement of the content and expression of LHCII *b* genes in the thylakoid membrane by nTiO_2_, promoting light absorption in chloroplasts [40]. In addition, ribulose-1,5-biphosphate carboxylase/oxygenase (RuBisCO) is also very sensitive to nTiO_2_, and increases in the rate of photosynthesis by nTiO_2_ can be attributed to RuBisCO activase acceleration facilitating the carboxylation [26]. Thus, higher photosynthesis can result in an increased supply of photoassimilates in leaves and better growth. Additionally, the stimulatory effect of nTiO_2_ in nutrient uptake and use efficiency can also be related to the increase of pigment levels and photosynthesis. nTiO_2_ improves nitrate reductase activity, increasing nitrogen assimilation and therefore increasing amino acids and protein production and growth [41]. Concerning the effects of nTiO_2_ on plant oxidative stress and antioxidant responses, it is described that these NM can act as pro-oxidants and antioxidants in modulation ROS signaling [42]. The changes in ROS level induced by nTiO_2_ are mostly associated with alterations in chloroplast function, one of the sites of major ROS production centers in plants [42]. In turn, it was also demonstrated that nTiO_2_ induces the activation of the biosynthesis of antioxidants, such as vitamin E [43].

The beneficial effects of nZnO in several plant processes, such as photosynthesis and the antioxidant system, are related to the putative increase of Zn availability and/or to the molecular effects of these NM [44,45]. Zn is an essential element that acts as a cofactor of a large number of key enzymes (e.g., SOD, carbonic anhydrase, and glutathione dehydrogenase), and it is involved in the metabolism of carbohydrates and proteins [46]. nZnO acts at the pigment synthesis level, promoting carotene and chlorophyll biosynthesis [47]. Moreover, these NPs can strengthen the plant vascular system, particularly the metaxylem tissues, improving the nutritional status [44]. Additionally, nZnO is described to modulate many genes and transcription factors (e.g., *ARP*, *MPK4*, *MKK2*, *SKRD2*, *MYC*, *bHLH*, *EREB*, *HsfA1a*, *R2R3MYB,* and *WRKY1*) associated with physiological, hormonal, and developmental responses, and abiotic stress tolerance [44,48]. Moreover, a recent study highlights that nZnO can induce epigenetic modifications, downregulating the histone deacetylases gene *(HDA3)* [44]. Furthermore, nZnO also modulates the transcription of genes of the antioxidant system, leading to a protective response by increasing the activity of several antioxidant enzymes [49].

Despite the evident advantage, and the potential to revolutionize the actual agricultural system, metal-based NM also have some ecotoxicological implications, as they may show a certain level of toxicity for plants [18,50,51,52], fungi, algae, and microorganisms [7,53]. Concerning the phytotoxicity of nTiO_2_ [54,55,56,57] and nZnO [58,59,60,61], it is frequently associated with higher doses; nevertheless, other factors such as plant species [62,63,64], exposure period [65,66,67], and crystalline phase in the case of nTiO_2_ [63,68] also play a role.

## 3. The Potential of nTiO_2_ and nZnO in Increasing Abiotic Stress Tolerance to Plants

Abiotic stresses are environmental factors that can limit plant growth, development, and productivity, and global climate change scenarios have contributed substantially to the intensification of these factors [69]. Therefore, strategies that reduce their adverse impact on plants need to be implemented to increase the resilience of plants to stress conditions. Among the several strategies adopted to mitigate the negative effects of abiotic stresses in plants, nanotechnology–particularly the use of NM, mostly NP—is one of the most promising [11].

Several metal-based NM, such as nTiO_2_ and nZnO, have been extensively studied in the last years due to their environmentally favorable use in agriculture, particularly in the promotion of plant growth and their protective role under stress conditions [39]. These studies have been mostly conducted in plants exposed to drought and salinity, since these abiotic stresses are the most common and produce a stronger impact on plant productivity [70,71,72]. The beneficial effects of the application of nTiO_2_ and nZnO in plants exposed to other abiotic stresses, such as high and low temperature [11,73], were less studied. For instance, under cold stress, nTiO_2_ foliar application (5 mg L^−1^) in *Cicer arietinum* increases the antioxidant enzymes activity, RuBisCO and phosphoenolpyruvate carboxylase, and the levels of pigments [74,75]. Moreover, these NPs reduce H_2_O_2_ content and membrane damage. The same NM applied in *Lycopersicon esculentum* (nano-anatase with 16 nm; 0.05, 0.1 and 0.2 g L^−1^) exposed to heat stress enhanced photosynthesis and promoted stomatal opening [76]. In wheat plants under heat conditions, foliar application of nZnO (10 ppm, size 80 nm) increased the antioxidant enzymes activity (e.g., SOD, CAT, GST, and peroxidase) and reduced the levels of lipid peroxidation [77].

### 3.1. Drought and Salinity

Climate changes have contributed to the increase of global drought, changing the precipitation patterns and increasing the periods without or with low precipitation [69]. Drought is therefore considered one of the most natural hazards, with important consequences in the agriculture sector and food security [70]. For instance, the occurrence of drought events in the European Union (particularly in southern and western parts) resulted in annual agriculture economic losses of around 10% [71]. Furthermore, global soil salinization is increasing due to climate change [72]. Intensive farming together with low-quality irrigation water and poor drainage have strongly contributed to soil salinization [78]. Currently, around 62 million hectares of the world’s irrigated area suffer from salinity, and this situation will be aggravated, particularly in the arid and semi-arid regions [79]. Therefore, drought and salinity are major global concerns and key factors that decline plant performance, yield, and productivity [78,80].

A common feature of these abiotic stresses is that they affect one of the most important key processes in plants—photosynthesis—reducing photosynthetic reactions and pigments levels but increasing the production of reactive oxygen species (ROS) leading to oxidative stress [72]. To control the levels of ROS and oxidative damages, plants can activate the antioxidant system, composed of enzymatic (e.g., superoxide dismutase (SOD), catalase (CAT), peroxidase (POD), and ascorbate peroxidase (APX)), and non-enzymatic (e.g., glutathione, ascorbate (AsA), and flavonoids) antioxidants [51]. In addition, plants can develop stress tolerance mechanisms to avoid negative effects of stress; nevertheless, they vary between species and depend on the intensity and duration of the stress event [72].

The use of stress mitigation agents to alleviate the negative impact of these abiotic stresses can also be an affordable strategy to maintain plant growth and productivity. Therefore, the search for new stress mitigation strategies that ensure food and nutritional security under the rising global population has increased. Several metal-based NM have been investigated and their potential to mitigate drought and salt stress adverse effects demonstrated in several works in different species (e.g., [28,39]). NP application in drought and salt-stressed plants has been reported to increase the availability of water in leaves and to promote growth (above and below-ground), biomass production, nutrient uptake, carbohydrate accumulation, and photosynthesis (Table 1 and Table 2). NM can also modulate phytohormone and osmolyte levels under drought and salinity conditions and contribute to the reduction of oxidative stress by the upregulation of the antioxidant battery (Table 1 and Table 2). Additionally, under salt stress conditions, NP can help to regulate ion balance, reducing Na^+^ toxicity and increasing the uptake of K^+^ in plants [28].

#### 3.1.1. Ameliorative Effects of nTiO_2_ in Plants Grown under Drought or Salinity Stress

nTiO_2_ is one of the most studied NPs, with applications in several areas, such as pharmaceutical, medicinal, industrial, and agricultural fields [39]. Most of the benefits of the application of these NM in plants, particularly at the photosynthesis level under both optimal and abiotic stress conditions, are related to the photocatalytic properties of nTiO_2_ [81]. Within the three crystalline structures of nTiO_2_ (anatase, rutile, and brookite), anatase exhibits the highest catalytic activity. The several advantages of nTiO_2_ application in plant species exposed to drought and salt stress conditions are summarized in Table 1 and Table 2.

Concerning drought, several studies have been conducted with wheat plants treated with nTiO_2_. Faraji and Sepehri [82,83] reported several positive effects in a controlled experiment using different nTiO_2_ concentrations for seed priming or soil amendment, respectively, before water deficit treatments (Table 1). These authors described that seed priming with nTiO_2_ promoted wheat shoot and root length and fresh weight, and soil amendment increased leaf water availability (relative water content—RWC) despite the higher stomatal conductance and transpiration and photosynthetic pigment levels (chlorophylls and carotenoids). Furthermore, in the same species and adding nTiO_2_ in pot soil, Mustafa et al. [81] demonstrated that they modulated the levels of hormones (increase of IAA and GA), proline, and carbohydrates under drought stress conditions. The wheat root length and nutrient uptake (K and P) were also improved by this NP under stress [81]. In maize plants, Karvar et al. [84] reported that nTiO_2_ foliar application increased the leaf RWC, F_v_/F_m_, carotenoids, chlorophylls, proline, soluble protein, and grain yield when plants were under drought. Besides photosynthesis, the production of secondary metabolites is also improved by the application of nTiO_2_ under drought conditions. Moreover, the enzymatic antioxidant system is also activated by nTiO_2_ in response to drought [39]. The activity of CAT, APX, and POD increased in basil, wheat, and maize plants treated with nTiO_2_ [81,82,84,85], reducing the levels of oxidative stress by decreasing the production of H_2_O_2_ and lipid peroxidation [82].

In the case of salt stress, the benefits of nTiO_2_ application are very similar to drought. In a study conducted by Sheikhalipour et al. [86], the nTiO_2_ foliar treatment of stevia plants exposed to different levels of salinity (50 mM and 100 mM NaCl) improved leaf availability (RWC), chlorophylls and carotenoids levels, and photosynthesis (net CO_2_ assimilation rate and F_v_/F_m_), leading to higher plant height and weight. At increased levels of salinity (180 mM), faba bean plants also respond to foliar nTiO_2_ treatment, increasing the levels of photosynthetic pigments, sugars, and proline, resulting in an improvement of growth [42]. nTiO_2_ application also ameliorated oxidative stress, activating several antioxidant enzymes (SOD, CAT, APX, and CAT) and reducing the levels of lipid peroxidation and H_2_O_2_ production [42,86]. In barley plants exposed to high levels of salinity (100 and 200 mM), nTiO_2_ application in soil improved photosynthesis (net CO_2_ assimilation rate, stomatal conductance, and transpiration rate) and chlorophyll and proline levels [87]. Moreover, barley leaf relative water content (RWC) and root length increased, as well as the activities of CAT and SOD, which contributed to the decrease of lipid peroxidation under salinity [87].

**Table 1:** Effects of nTiO_2_ on plants under drought conditions.

Stress Conditions	nTiO_2_ Crystalline Phase_;_ Concentrations; Primary (PS) or Hydrodynamic Size (HS)	Plant Species	Application Method	Ameliorative Effects	Ref.
After seed-filling maintained at 50% FC	Anatase; 10, 100, and 500 mg L^−1^; PS 10–25 nm.	*Linum usitatissimum* L.	Three foliar applications at the initial seed-filling	Increased leaf carotenoids and seed protein	[88]
PEG-6000 solutions at −0.4 and −0.8 MPa	Not stated; 500 and 2000 mg L^−1^ PS 10–25 nm.	*Triticum aestivum* L.	Seed priming for 7 days	Increased shoot and root length, as well as fresh weight	[83]
75% and 50% FC for 6 weeks	Not stated; 500, 1000 and 2000 mg kg^−1^ soil; PS 10–25 nm.	*Triticum aestivum* L.	Soil amended	Increased leaf RWC, chlorophylls and carotenoids levels, and antioxidant enzymes (APX, CAT). Decreased lipid peroxidation and H_2_O_2_ production	[82]
45% FC for 15 days	Not stated; 20 and 40 mg kg^−1^ soil; PS 347–447 nm.	*Triticum aestivum* L.	Soil amended	Increased root length, hormone level (IAA and GA), proline and sugars. Improvement of the antioxidant enzymes (SOD, POD and CAT) and nutrient uptake (K and P)	[81]
105 and 140 mm evaporation from the class A evaporation pan (mm day^−1^)	Anatase; 50 and 100 mg L^−1^ PS 10–25 nm.	*Zea mays* L.	Foliar spray twice with an interval of 2 weeks (at the 4–6 leaf stage and 2 weeks after that)	Increased leaf relative water content, Fv/Fm, carotenoids, chlorophylls, proline, soluble protein and grain yield. Improvement of the activity of the antioxidant enzymes (SOD, APX and CAT)	[84]

**Table 2:** Effects of nTiO_2_ on plants under salinity conditions.

Stress Conditions	nTiO_2_ Crystalline Phase_;_ Concentrations; Primary (PS) or Hydrodynamic Size (HS)	Plant Species	Application Method	Ameliorative Effects	Ref.
180 mM NaCl for 28 days	Not stated; 160, 320 and 480 mg L^−1^; PS 20–78 nm.	*Vicia faba* L.	Two foliar applications, 3 and 10 days after NaCl treatments	Increased root and shoot length and dry weight. Improvement of the levels of proline, sugars, chlorophylls and carotenoids. Increased antioxidant enzyme activities (SOD, POD, APX and CAT). Decreased lipid peroxidation and H_2_O_2_ production	[42]
100 and 200 mM NaCl for 40 days	Anatase; 500, 1000 and 2000 mg kg^−1^; PS 10–25 nm.	*Hordeum vulgare* L.	Soil amended	Increased root length, leaf RWC, net CO_2_ assimilation rate, stomatal conductance, transpiration rate, chlorophyll and proline. Improvement of the antioxidant enzyme activities (APX and CAT). Decreased lipid peroxidation	[87]
50 and 100 mM NaCl for 2 weeks	Anatase; 100 and 200 mg L^−1^; PS 25 nm.	*Stevia rebaudiana* Bertoni.	Foliar spray for 3 times (during the growth period)	Increased root and shoot height, fresh and dry weight, leaf RWC, chlorophylls, carotenoids contents. Improvement of the net CO_2_ assimilation rate and F_v_/F_m_. Increased antioxidant enzyme activities (SOD, POD, APX and CAT). Decreased lipid peroxidation and H_2_O_2_ production	[86]

#### 3.1.2. Ameliorative Effects of nZnO in Plants Grown under Drought or Salinity Stress

nZnO has been widely used in several areas (e.g., cosmetic and medicine), but its higher popularity arises from its use in fertilizers and pesticides manufacture [89]. Zn is an important cofactor of several essential enzymes, and the benefits of nZnO application in plants are in part based on the increased availability of this nutrient to the plant, which leads to improvements on several metabolic pathways [45]. Several advantages of nZnO application in plant species have been reported under both drought and salt stress conditions (Table 3 and Table 4).

Concerning drought stress, positive effects of nZnO treatments (seed priming, soil amendment, or foliar application) on wheat, cucumber, and aubergine plants were reported, increasing water availability, both stages of photosynthesis, light-dependent and independent reactions, and photosynthetic pigments [80,90,91,92]. nZnO stimulated carbohydrate (e.g., leaf sugar levels) and amino acid (e.g., proline, glycine betaine, and free amino acids) metabolism and increased shoot and root growth (length, fresh and dry weight) in maize and cucumber plants [80,93]. Furthermore, nZnO seems to induce a strong boost of the antioxidant system, upregulating the expression and activity of several antioxidant enzymes (SOD, POD, APX, CAT, glutathione reductase (GR), dehydroascorbate reductase (DHAR), monodehydroascorbate reductase (MDHAR), and phenylalanine ammonia lyase (PAL) activity) and non-enzymatic antioxidants (ascorbate, glutathione, total phenols, and flavonoids) [80,91,93] leading to lower oxidative stress due to less lipid peroxidation, membrane leakage, and O_2_**^−^**^•^ and H_2_O_2_ accumulation under drought stress conditions [80,90,91,93]. A study conducted with sorghum plants also showed that nZnO application in soil improves grain yield [37] and in wheat grains enhances nutrient levels under drought conditions [92].

**Table 3:** Effects of nZnO on plants under drought conditions.

Stress Conditions	nZnO Concentrations; Primary (PS) or Hydrodynamic Size (HS)	Plant Species	Application Method	Ameliorative Effects	Ref.
30% of total moisture for 3 days.	Not stated	*Triticum aestivum* L.	Seed priming for 4 h	Increased leaf RWC, chlorophyll and carotenoids levels, and antioxidant enzyme activities (SOD and CAT). Decreased lipid peroxidation	[91]
40% field capacity	1, 3 and 5 mg L^−1^; PS 18 nm	*Sorghum bicolor*	Soil amended	Increased grain yield	[37]
40% field capacity for 210 days	2.17 mg kg^−1^; not stated	*Triticum aestivum* L.	Soil amended	Increased chlorophyll content and grain nutrient	[92]
6 days at 45% (soil water content).	100 mg L^−1^; PS 20 nm	*Zea mays* L.	Seed priming	Increased root and shoot height, fresh and dry weight, as well as sugars, protein, amino acids (tryptophane) and proline. Improvement of antioxidant enzyme activities and gene relative expression (SOD, POD, APX and CAT). Decreased H_2_O_2_ production	[93]
12 days	25 and 100 mg L^−1^; PS 50 nm	*Cucumis sativus* L.	Foliar application 3 time a week, for two weeks	Increased shoot fresh and dry weight, root dry weight and length, leaf RWC, chlorophylls, carotenoids, protein content, net CO_2_ assimilation rate, stomatal conductance, transpiration rate, intercellular CO_2_ concentration, F_v_/F_m_, qP and Φ_PSII_. Accumulation of proline, glycine betaine, free amino acids, and sugars. Improvement of antioxidant enzyme activities (SOD, POD, APX, CAT, GR, DHAR and MDHAR) and PAL activity. Increased total phenols, flavonoids, ascorbate (AsA) and glutathione. Decreased O_2_^−•^ and H_2_O_2_ production, lipid peroxidation, electrolyte leakage and NPQ	[80]
60% of crop evapotranspiration (ETc) for 5 months.	50 and 100 mg L^−1^; not stated	*Solanum melongena* L.	Foliar application 2 times	Increased leaf RWC and F_v_/F_m_. Improved membrane stability	[90]

Under salinity conditions (30–150 mM), different nZnO applications (soil amendment or foliar spray) induced positive effects in potato, tomato, and flax plants (Table 4), with improvements at the shoot and root growth attributes (length, fresh and dry weight), leaf area, photosynthetic parameters (including both light-dependent and independent reactions), chlorophyll, protein, and proline [94,95,96]. In these species, nZnO treatment increased leaf nutrient uptake and stimulated the antioxidant system (antioxidant enzymes), leading to lower oxidative stress (reduction of lipid peroxidation, O_2_**^−^**^•^ and H_2_O_2_ production). In addition, Mahmoud et al. [96] verified an increase of the RWC and gibberellic acid (GA) levels in salt-stressed potato plants. Furthermore, nZnO foliar application stimulated the carbohydrate and amino acid metabolism in mango plants watered with salinized drainage water [97]. These authors also reported an enhancement of nutrient levels and antioxidant capacity (antioxidant enzymes SOD, POX, and CAT).

**Table 4:** Effects of nZnO on plants under salinity conditions.

Stress Conditions	nZnO Concentrations; Primary (PS) or Hydrodynamic Size (HS)	Plant Species	Application Method	Ameliorative Effects	Ref.
Irrigated in the beginning with 150 mM NaCl	20, 40 and 60 mg L^−1^; PS 21.3 nm	*Lupinus termis* Forssk.	Seed priming for 12 h	Increased proline, protein and free amino acids, sugars, chlorophylls and carotenoids, total phenols and AsA. Improved the antioxidant enzyme activities (SOD, POD, APX and CAT). Decreased lipid peroxidation	[89]
Irrigated in the beginning with 108 mM NaCl	10 mg L^−1^; PS 30 nm	*Brassica napus* L.	Three foliar applications at 50, 65, and 80 days after sowing	Increased carotenoids, proline and sugars. Improved the activity of antioxidant enzymes (SOD, POD, and CAT), and the pool of ASA and total phenolic compounds. Decreased H_2_O_2_ production, lipid peroxidation and membrane leakage	[98]
Salinized drainage water	50, 100 and 150 mg L^−1^; PS < 100 nm	*Mangifera indica* L.	Two foliar applications (full bloom and 1 month after)	Enhanced leaf NPK content, total carbohydrates and proline. Increased the activities of the antioxidant enzymes SOD, POX, and CAT	[97]
<30 ds/m NaCl	12, 15 and 20 mg L^−1^; PS 4.50–5.80 nm	*Solanum tuberosum* L.	Soil amended, 15 days before planting, and 20, 35, 45 and 70 days after planting	Increased plant height, fresh and dry weight, and leaf RWC. Improved the net CO_2_ assimilation rate, stomatal conductance, intercellular CO_2_ concentration and WUE. Increased the levels of chlorophyll, proline, phytohormones (GA) and leaf nutrients (N, P, K, Ca, Na, Zn and B). Decreased the transpiration rate and the levels of ABA	[96]
150 mM NaCl	10, 50, 100 mg L^−1^; not stated	*Lycopersicon esculentum* Mill.	Soil amended at the time of transplanting (15 days after sowing)	Increase the shoot and root fresh and dry weight, and length, leaf area, protein content, proline, and chlorophyll. Improved the net CO_2_ assimilation rate, stomatal conductance, transpiration rate and intercellular CO_2_ concentration. Improved the activity of the antioxidant enzymes (SOD, POD and APX).	[94]
150 mM NaCl applied 30 days after sowing	50 mg/L; not stated	*Linum usitatissimum* L.	Foliar application 60 days after sowing	Increased shoot fresh and dry weight, root dry weight and length, leaf area, and leaf nutrients (C, K and Ca). Improved net CO_2_ assimilation rate, stomatal conductance, intercellular CO_2_ concentration, WUE, F_v_/F_m_, qP and Φ_PSII_. Increased the levels of chlorophylls, proline, carbohydrates, NR, carbonic anhydrase, and the activity of antioxidant enzymes (SOD, POD, and CAT). Decreased the O_2_^−^ and H_2_O_2_ production, lipid peroxidation.	[95]
150 mM NaCl for 7 days	25, 50 and 100 mg L^−1^; not stated	*Brassica napus* L.	Seed priming for 8 h	Increased sugar, soluble protein and SOD activity	[99]

nZnO foliar application improved the levels of carotenoids, proline, sugars, and antioxidants (enzymes, ascorbate, and total phenolic compounds) in salt-stressed rapeseed plants [98]. These authors reported that nZnO decreased the levels of oxidative stress (H_2_O_2_ production, lipid peroxidation, and membrane leakage) in this species caused by salinity (108 mM). In Lupinus exposed to 150 mM NaCl, seed priming with nZnO increased protein, sugars, free amino acids, including proline, and pigments levels, as well as antioxidant enzyme activities (SOD, POD, APX and CAT) and non-enzymatic antioxidants (total phenols and ascorbate) resulting in lower levels of lipid peroxidation [89]. Furthermore, rapeseed seeds primed with nZnO lead to improved levels of sugars, soluble protein, proline, and increased activity of SOD under 150 mM NaCl [99]. These authors also verified that these NPs upregulated the gene *BnPER* (peroxiredoxin antioxidant family gene) in priming seeds, contributing to oxidative damage reduction under salinity.

### 3.2. Environmental Contaminants

Climatic stresses combined or separated with xenobiotics induce major damage in sensitive crops, making them major threats to food security and the financial safety of farmers [100]. Soil contamination by metals, in particular heavy metals, is an environmental problem worldwide, with a negative impact on agriculture as a consequence of their phytotoxicity, low mobility, non-biodegradable nature, and high persistence [101,102]. Moreover, the contamination of soils by metals is a health problem due to the trophic transfer in the food chain and the bioaccumulation and biomagnification of metals [103].

The consumption of crops contaminated with metals causes risks to human health and animals, as some of these elements are not essential to humans (e.g., Pb, Cd, As, Hb, Al), and others, despite having functional roles in humans (e.g., Zn, Fe, Mn, Mg, Cr, Ni, Cu, Mo, Se), can have adverse health outcomes at high doses. Some of the putative effects may include alterations in reproductive health [104], impacts on the nervous system, the induction of carcinogenesis, oxidative stress, and loss of cellular functions [105,106].

In crops, multiple effects have been described to be induced by metal exposure, frequently dependent on the dose, exposure period, crop species and/or genotype, and soil physicochemical characteristics (e.g., pH) [107]. Impairments in plant morphology, physiology, biochemistry, and adjustments in plant metabolism are transversal to a high number of metal contaminants, as well as their cytotoxicity and genotoxicity [107,108].

Some of the metals of great environmental concern include chromium (Cr), cadmium (Cd), lead (Pb), arsenic (As), and aluminum (Al). Cr—in particular, its hexavalent form (VI) [107]—can reduce the germination rate [109], impair root development, reduce plant biomass/growth and yield [109,110,111], damage membranes [110], impair photosynthesis [111,112,113], and induce chlorosis and necrosis [108,114], oxidative stress [110,113,115], and ultrastructural changes [116], as well as DNA damage [107,117]. Cd can also inhibit photosynthesis [118,119], induce reactive oxygen species (ROS) overproduction and oxidative stress [120], and induce genotoxicity and cytotoxicity [121]. Pb is described as being able to impair germination and plant growth [122], even under low concentrations [123], decrease the net photosynthetic rate and effective PSII photosynthetic efficiency [124], impair the Calvin cycle [125], and induce DNA damage [126,127,128] and antioxidant response due to redox homeostasis loss [128,129]. Al, which represents 7% of the soil matter of the Earth’s crust, can severely impair crop development and yield in acidic environments [130,131], can change root ultrastructure and development [132,133,134,135], induce nutrient imbalances by limiting the availability of minerals such as Mg, Ca, and K [136,137,138], negatively affect photochemical and non-photochemical phases of photosynthesis [139,140,141], and can increase ROS production [142,143,144,145]. Arsenic (As) is a persisting metalloid in the environment and promotes ROS production and oxidative damage [146], including cell membrane and DNA damages, and alters photosynthesis and nutrient supply [147]. Likewise, copper (Cu), despite not being a xenobiotic due to its role as a micronutrient, may impair plant growth and photosynthetic processes [148], alter root ultrastructure [149], and induce oxidative stress [150].

Both metallic and metal oxide nanomaterials, including SiO_2_, TiO_2_, Fe_2_O_3_, Fe_2_O_4_, ZnO, Mn_3_O_4_, and CeO_2_, have been described to mitigate, at some level, the toxicity of metals in plants. Nevertheless, the benefits of these materials are frequently linked with low doses, whereas higher concentrations are more prone to induce toxicity [100,151], highlighting the need to optimize the dose when it is intended to use this kind of materials in agriculture.

#### 3.2.1. Ameliorative Effects of nTiO_2_ in Plants Exposed to Environmental Contaminants

nTiO_2_ has been described, during the last half decade, to be able to ameliorate the toxic effects of several environmental contaminants, including Cd, Cu, Pb, Al, Sb, As, 2,4-dichlorophenoxyacetic acid, and tetracycline, although most works focus on Cd toxicity (Table 5 and Table 6). In the majority of works, it is not stated whether the crystalline phase of nTiO_2_ is considered, making it impossible to establish a relationship between the crystalline phase and the effects reported.

Concerning the nTiO_2_ application method, two main routes are used: (1) via roots in a solid matrix, by mixing nTiO_2_ powder [152] with the soil/substrate or by spiking with nTiO_2_ suspensions [153,154,155,156], or in hydroponic systems using NP suspensions [157,158,159,160,161]; (2) via leaves by foliar spray with nTiO_2_ suspensions [157,162,163]. Besides, seed priming was analyzed by Sardar et al. [164], whereas Dai et al. [165] applied nTiO_2_ during the seedling stage in petri-dishes with moistened paper, and Katiyar et al. [166] treated the seeds and seedlings with nTiO_2_ suspensions, both simultaneously with the contaminant.

**Table 5:** Ameliorative effects of nTiO_2_ application against the phytotoxicity of Cadmium (Cd).

Salt; Concentration	nTiO_2_ Crystalline Phase; Concentrations Used; Primary (PS) or Hydrodynamic Size (HS)	Plant Species	Application Method	Ameliorative Effects	Ref.
CdCl_2_; 50 mg kg^−1^	Not stated; 40, 80, 160 mg L^−1^; PS < 100 nm	*Coriandrum sativum* L.	Seed priming (24 h)	Decreased Cd uptake and improved germination rate, plant growth and biomass; increased pigment contents; improved gas exchange parameters; increased CAT, SOD and APX activity; increased proline level; decreased MDA content and electrolyte leakage; improved seed yield	[164]
Cd(NO_3_)_2_; 13.95 mg kg^−1^	Not stated; 100–1000 mg k^−1^; PS 15–40 nm	*Trifolium repens*	In soil (80 d)	Increased plant length and biomass	[153]
CdCl_2_; 10 mg kg^−1^	Not stated; 100, 200 mg L^−1^; PS 100 nm	*Vigna unguiculata*	Foliar spray in 21 days-old plants	Increased chlorophyll *b*; decreased in Cd uptake and translocation; MDA decrease; stimulated the antioxidant enzyme activity; increased Zn, Mn and Co in seeds	[162]
1.03, 2.46, 5.06 mg kg^−1^	Not stated; 50, 100, 500 mg kg^−1^; PS 20–40 nm	*Oryza sativa* L.	In soil (30, 60, 90 d)	Increased the plant height in tillering and booting growth stages; decreased MDA content and the activity of antioxidant enzymes, mostly when plants were treated with the higher doses of TiO_2_	[152]
50 µM	Anatase; 100; 250 mg L^−1^; PS 6.5 nm; HS 310–421 nm in foliar spay; HS 700–1880 nm in hydroponics	*Zea mays* L.	Foliar spray in 19 days-old plants (for 14 days evenly) and hydroponic system	Foliar spray: increased the membrane integrity (250 mg L^−1^); decreased Cd content in roots (100 mg L^−1^) and shoots (both); downregulated amino acid metabolic pathways Hydroponics: increased membrane integrity (250 mg L^−1^); decreased Cd content in roots (250 mg L^−1^); upregulated carbohydrates metabolic pathways	[157]
8.5 mg L^−1^	Sodium dodecyl benzene sulfonate-coated and uncoated nTiO_2_; 100, 200, 500, 1000 mg L^−1^; HS 260–350 nm	*Triticum aestivum L.*	Seedlings In petri dishes with moistened filter paper (5 days)	The highest doses increased root length	[165]
CdCl_2_; 10, 20 mg L^−1^	Not stated; 10, 100, 1000 mg L^−1^; PS 18–166 nm	*Oryza sativa* L.	Hydroponic system (10 days)	Stimulated plant growth; decreased Cd uptake; stimulated the net photosynthetic rate and chlorophyll content; decreased the MDA and modulated the antioxidant response	[158]
CdCl_2_; 100 mg kg^−1^	Not stated; 100, 200, 300 mg kg^−1^; PS < 100 nm	*Glycine max* L.	In soil (30–60 days after sowing)	Decreased proline content; increased protein content; increased chlorophyll *b* content	[154]
Contaminated soil; 7.86 mg kg^−1^	5, 10, 20, 30 mg L^−1^; PS 20–30 nm;	*Oryza sativa* L.	Foliar spray at 26, 33 and 40 d after sowing	Stimulated plant growth; promoted gas exchange; increased chlorophyll contents; decreased MDA, electrolyte and H_2_O_2_ contents in both roots and leaves; stimulated antioxidant enzyme activities; decreased Cd accumulation and translocation	[163]

**Table 6:** Ameliorative effects of nTiO_2_ application against the phytotoxicity of several environmental contaminants.

Contaminant	Salt; Concentration	nTiO_2_ Crystalline Phase; Concentrations Used; Primary (PS) or Hydrodynamic Size (HS)	Plant Species	Application Method	Ameliorative Effects	Ref.
Cu	CuSO_4_·5H_2_O; 1, 2 mg L^−1^	Anatase; 10 mg L^−1^; HS 374 nm (1 h in suspension); HS 1064 nm (48 h in suspension)	*Glycine max* L.	Hydroponic system (6 days)	Decreased the translocation factor of Cu	[159]
Pb	Pb(NO_3_); 10 mg kg^−1^	P25; 5 mg kg^−1^; HS ~130 nm	*Lactuca sativa* L.	In soil (12 days)	Decreased the relative membrane permeability; increased pigment contents; promoted gas exchange, including the net photosynthetic rate	[155]
Al	AlCl_3_·6H_2_O; 50 mg kg^−1^	P25; 5 mg kg^−1^; HS ~130 nm	*Lactuca sativa* L.	In soil (12 days)	Decreased the relative membrane permeability; promoted gas exchange; increased the effective efficiency of photosystem II	[155]
Sb	K_2_H_2_Sb_2_O_7_⋅4H_2_O; not stated	Not stated; 100–250 mg kg^−1^; PS 15–40 nm	*Sorghum bicolor*	In soil (80 days)	Increased the germination rate	[156]
As	Sodium arsenate; 10 µmol L^−1^	Not stated; Chemical NPs: 2500 mg L^−1^; HS 64.3 nm. Green NPs: 1000 mg L^−1^; HS 53.2 nm	*Vigna radiata* L.	Seeds treated prior germination and during the germination period	Increased biomass and seedling length; decreased H_2_O_2_ and MDA contents; increased the protein content, and gene expression of SOD and CAT	[166]
2,4-Dichloro phenoxyacetic acid	1000 µM	Not stated; PS < 100 nm; HS 260 nm	*Azolla pinnata* R.Br	Pre-treatment with TiO_2_ (3 days) followed by exposure to 2,4-D in a hydroponic system	Modulated K, N, P accumulation; increased biomass; increased the activity of the enzyme invertase; promoted the nitrogen metabolism	[167]
Tetracycline	1, 5, 10 mg L^−1^	Rutile; 40, 100, 200 mg L^−1^; PS 5–15 nm	*Arabidopsis thaliana* L.	Hydroponic system (12 days)	Increased fresh biomass (40 mg L^−1^); altered the activity of antioxidant enzymes; changed the expression of genes encoding GST, MDHAR, GR, SiR, APR, APT	[161]
	5–20 mg L^−1^	Anatase; 500, 1000, 2000 mg L^−1^; PS 10–25 nm	*Oryza sativa* L.	Hydroponic system (10 d)	Increased shoot and root biomass; decreased tetracycline content in shoots and roots; modulated nutrient accumulation; decreased antioxidant enzymes activity; showed antagonist effect with tetracycline	[160]

Besides the application methods, the NP concentrations and size used, as well as the treatment period, are also very diverse, with concentrations ranging from 50 to 500 mg kg^−1^ in soil experiments, 10 to 2000 mg L^−1^ in hydroponic systems, and 10 to 200 mg L^−1^ in foliar application, whereas the treatment period may range from 24 h to 90 days (Table 5 and Table 6). Nevertheless, despite these differences, it is evident that nTiO_2_ application may alleviate toxic symptoms induced by several contaminants.

Under Cd, soil amendment with nTiO_2_ improved several physiological attributes in white clover [153], rice [152], and soybean [154]. In white clover, nTiO_2_ (500 mg kg^−1^) stimulated plant growth with the increase of plant length and biomass [153]. Similarly, nTiO_2_ (500 mg kg^−1^) application in rice improved plant growth and also decreased MDA content simultaneously with the decrease of CAT, SOD, and POD activity [152]. In soybean, nTiO_2_ (100–300 mg kg^−1^) increased the protein and chlorophyll *b* contents and decreased the proline content [154]. Furthermore, in rice, but using a hydroponic system, nTiO_2_ treatments (10–1000 mg L^−1^) showed some potential to stimulate plant growth, reducing the MDA content, modulating the antioxidant response, altering the levels of some phytohormones (indole-3-acetic acid, isopentenyl adenosine, methyl jasmonate, and zeatin riboside), and stimulating photosynthesis by increasing the net photosynthetic rate and the chlorophyll content [158]. In addition, and in contrast to what was described by Zhang et al. [152], in this case, the Cd uptake decreased. Root application of nTiO_2_ using a hydroponic system in maize also decreased Cd content in roots (250 mg L^−1^), likewise increasing the membrane integrity and upregulating the carbohydrate metabolic pathways [157].

The foliar spray with nTiO_2_ also shows potential in mitigating Cd phytotoxicity. In cowpea [162] and rice [157,163], leaf application of nTiO_2_ decreased Cd uptake and increased membrane integrity. In both species, the increment of pigment levels (Chl *b* in cowpea and Chl a, *b* and carotenoids in maize) was also reported, as well as the stimulation of the antioxidant response by increasing the activity of antioxidant enzymes [162,163]. Besides the pigment levels, nTiO_2_ also stimulated the photosynthesis in maize by improving the gas exchange parameters, including the net photosynthetic rate, transpiration rate, and stomatal conductance, which may have contributed to the observed stimulation of plant growth [163]. Interestingly, the mitigating effects observed by Rizwan et al. [163] were induced despite using 10 times lower concentrations of nTiO_2_ than Ogunkunle et al. [162] and Lian et al. [157].

At the seedling stage, sodium dodecyl benzene sulfonate-coated and uncoated nTiO_2_ (1000 mg L^−1^) increased the root length of wheat exposed to 8.5 mg L^−1^ of Cd [165]. Finally, the priming of coriander seeds [164] with nTiO_2_ (40, 80 and 160 mg L^−1^) also showed promising results and a potential strategy to mitigate Cd phytotoxicity (50 mg kg^−1^) once: it enhanced the germination rate, plant growth, and biomass; increased the pigment levels (80 mg L^−1^); stimulated the non-photochemical phase of photosynthesis by increasing the intercellular CO_2_ content, stomatal conductance, transpiration rate, and net photosynthetic rate; stimulated an antioxidant response by increasing the activity of the enzymes CAT, SOD and APX, and the proline content; improved membrane integrity; improved the seed yield; and decreased Cd uptake.

Under Cu and using a hydroponic system, nTiO_2_ (10 mg L^−1^) decreased the translocation factor of Cu in soybean [159]. In lettuce grown in soil, Mariz-Ponte et al. [155] reported ameliorative effects induced by nTiO_2_ (5 mg kg^−1^) amendment in the presence of Pb or Al. In both cases, nTiO_2_ decreased the relative membrane permeability and promoted the intercellular CO_2_ content, stomatal conductance, transpiration rate, and net photosynthetic rate. Furthermore, under Pb, nTiO_2_ also increased the pigment content (chlorophyll *a*, *b* and carotenoids), whereas in the presence of Al, it enhanced the effective efficiency of photosystem II [155]. nTiO_2_ (2500 mg L^−1^) also showed positive effects to mitigate As effects in mung bean by upregulating the expression of antioxidant enzymes (SOD and CAT), which may have conferred greater protection against oxidative damages, as supported by the decrease of MDA content and ROS levels (H_2_O_2_ and O_2_**^−^**^•^) [166].

Besides metal contaminants, nTiO_2_ was able to mitigate some effects of 2,4-dichlorophenoxyacetic acid (2,4-D), a systemic herbicidal, and tetracycline, an antibiotic widely used in agriculture and livestock industries. In the case of 2,4-D, nTiO_2_ increased the biomass, promoted the accumulation of N and K, despite reducing P content, upregulated the activity of soluble invertase to values closer to the control (despite decreasing the amount of cell wall bounded invertase), and promoted the nitrogen metabolism by increasing nitrate reductase and glutamine 2-oxoglutarate amino transferase activities [167]. Concerning the tetracycline, nTiO_2_ application alleviated the negative effects on plant/pod biomass in *Arabidopsis* [161] and rice [160] grown in hydroponics. Furthermore, in both species, nTiO_2_ modulated the antioxidant response, altering the activity of antioxidant enzymes, and in *Arabidopsis*, it upregulated the expression of adenyltransferase (APT), adenosine-5′-phosphosulfate reductase (APR), and sulfite reductase (SiR) in the roots. It is worth mentioning that in rice, nTiO_2_ significantly decreased the levels of tetracycline in shoots and roots and altered the nutrient content, showing a trend to increase P, S, and Zn [160].

Taking into consideration all the works presented in Table 5 and Table 6, it seems that nTiO_2_ has the capability to decrease metals and non-metals contaminants uptake and translocation to shoots when the NPs are applied as suspensions to the leaves/seeds or are added to a nutritive solution together with the contaminant (hydroponic system). When nTiO_2_ is applied to the soil, it looks to promote metal uptake, nevertheless without increasing its toxicity. In fact, an overall mitigation of toxic symptoms was observed. In both cases, an antagonistic effect is reported, as was particularly stated by Mariz-Ponte et al. [155] and Ma et al. [160], reducing metal phytotoxicity and enhancing plant performance. The mechanism underlying these results may be related to a reduction in contaminant bioavailability due to their immobilization by nTiO_2_ [10,156,160].

#### 3.2.2. Ameliorative Effects of nZnO in Plants Exposed to Environmental Contaminants

The studies available using nZnO as a strategy to cope with the adverse effects of environmental contaminants in plants, despite being limited and fractionated, unveil their potential to improve plant physiology under stress conditions. Similar to nTiO_2_, nZnO was mostly explored to ameliorate Cd toxic effects, despite a couple of studies existing with As, Pb, Cu, and Co (Table 7 and Table 8). Most of the works focus on plant growth, ROS production, oxidative stress, and the antioxidant response, and show the capability of nZnO to modulate those physiological processes under metal stress.

Cd phytotoxicity was alleviated in rice [168,169], maize [170], and wheat [73,171] plants sprayed with nZnO suspensions (25–100 mg L^−1^). In all these species, plant growth improvements were described, such as plant length and biomass; promotion of gas exchange, including the net CO_2_ assimilation rate; an increase of pigment contents; and a decrease of Cd uptake and/or translocation. In addition, the stimulation of the antioxidant response with the upregulation of antioxidant enzymes (CAT, SOD, G-POX) [73,168,170,171] and proline [168] was reported, together with the reduction of ROS and MDA content and decrease of electrolyte leakage. Similar responses were obtained in wheat after seed priming with nZnO (25–100 mg L^−1^) [172] and when nZnO (25 mg L^−1^) was supplemented in a hydroponics system in Leucaena seedlings [61].

**Table 7:** Ameliorative effects of nZnO application against the phytotoxicity of Cadmium (Cd).

Salt; Concentration	nZnO Concentration; Primary (PS) or Hydrodynamic Size (HS)	Plant Species	Application Method	Ameliorative Effects	Ref.
CdCl_2_; 0.8 mM	50 mg L^−1^; not stated	*Oryza sativa* L.	Foliar spray at 30 to 35 days after sowing	Improved plant length and biomass; Higher chlorophyll index; improved the gas exchange, including the net CO_2_ assimilation rate; reduced ROS accumulation and MDA content; promoted CAT activity; increased proline levels; promoted essential nutrient uptake; decreased Cd accumulation	[168]
Contaminated soil; 7.67 mg kg^−1^	25, 50, 100 mg L^−1^; PS 20–30 nm	*Triticum aestivum* L.	Foliar spray at two, three, four and five weeks after sowing	Enhanced plant growth; increased grain dry weight; increased pigment content; decreased MDA, electrolyte leakage and H_2_O_2_ content; upregulated SOD and CAT activity; decreased Cd accumulation and transfer to shoots, and Cd bioavailability	[171]
7.86 mg kg^−1^	50, 75, 100 mg L^−1^; PS 20–30 nm	*Zea mays L*.	Foliar spray in 32 days-old plants	Increases shoot and root dry weight; enhanced photosynthetic pigment content and gas exchange related parameters; decreased MDA and membrane permeability; promoted CAT, APX and POD activity; decreased Cd accumulation	[170]
Contaminated soil; 7.38 mg kg^−1^	25, 50, 75, 100 mg L^−1^; PS 20–30 nm;	*Triticum aestivum* L.	Seed priming	Stimulated plant growth; promoted gas exchange; increased pigment contents; decreased electrolyte leakage; increased SOD and G-POX activities; decreased Cd content and bioavailability	[172]
Contaminated soil; 7.38 mg kg^−1^	50, 75, 100 mg L^−1^; PS 20–30 nm;	*Orysa sativa* L.	Foliar spray after 14, 21, 38 and 35 days after transplantation	Increased shoot length and dry weight, and root dry weight; increased chlorophyll *a* and *b* contents; stimulated gas exchange; decreased Cd uptake and translocation	[169]
Contaminated soil; 7.38 mg kg^−1^	25, 50, 75, 100 mg L^−1^; PS 20–30 nm;	*Triticum aestivum* L.	In soil; Foliar spray after 2, 4, 6 and 8 weeks after sowing	Promoted plant growth; increased grain dry weight; increased chlorophyll *a* and *b*, and carotenoid contents; stimulated gas exchange; electrolyte leakage decrease; SOD and G-POX activities increase; decreased Cd uptake and translocation, and Cd accumulation in grains	[73]
CdCl_2_; 50 mg L^−1^	25 mg L^−1^; PS 2–64 nm	*Leucaena leucocephala* (Lam.) de Wit	Hydroponic system (15 days)	Promoted plant growth; increased chlorophyll *a* and *b*, and carotenoid contents; increased total soluble protein levels; SOD, CAT and G-POX activities increase; decrease of DHA damage	[61]

In Leucaena, nZnO induced the enhancement of soluble protein and genomic alterations (presence of new DNA bands and/or absence of normal bands in the RAPD pattern of the exposed plants); nevertheless, in contrast to the previous works, in this case, it augmented the Cd accumulation [61]. Besides Cd, Venkatachalam et al. [61] also conducted the same experiment under Pb stress, with similar ameliorative results as those obtained for Cd. Under combined exposure of low Cd concentration (1 mg L^−1^) and high Pb (100 mg L^−1^), the amendment with polyvinylpyrrolidone-coated nZnO (100 mg L^−1^) decreased Cd content in shoots of cilantro, parsley, and spinach, whereas it increased the Pb levels in cilantro and did not affect the Pb content in parsley and spinach [173]. In roots, Sharifan et al. [173] described that Cd content decreased in parsley and spinach and that Pb was significantly reduced in all three species. The authors attributed the Cd and Pb mitigation to the adsorption of metals onto the nZnO surface, despite its overall significance possibly being affected by the nZnO surface charge plus the presence of roots exudates [173]. Additionally, nZnO altered the dynamic translocation and uptake of essential minerals such as Cu, Fe, and Zn: Fe increased in shoots of parsley and spinach; Zn increased in all species and both organs; and Cu decreased in cilantro shoots [173]. Furthermore, under combined stress, sunflower plants irrigated with heavy-metal-contaminated wastewater (mainly Cr, Cd, and Pb) showed improved performance when foliar sprayed with nZnO (60 mg L^−1^), as well as a decrease in metal content [174].

**Table 8:** Ameliorative effects of nZnO application against the phytotoxicity of environmental contaminants.

Contaminant	Salt	Salt Concentration	nTiO_2_ Crystalline Phase; Concentrations Used; Primary (PS) or Hydrodynamic Size (HS)	Plant Species	Application Method	Ameliorative Effects	Ref.
Pb	PbNO_3_	100 mg L^−1^	25 mg L^−1^; PS 2–64 nm	*Leucaena leucocephala* (Lam.) de Wit	Hydroponic system (15 d)	Promoted plant growth; increased chlorophyll *a* and *b*, and carotenoid contents; decreased MDA content; increased total soluble protein levels; SOD, CAT and G-POX activities increase; decrease of DHA damage	[61]
Cd + Cr + Pb	Irrigation with contaminated wastewater	3.1 mg kg^−1^ + 39.5 mg kg^−1^ + 14.4 mg kg^−1^	60 mg L^−1^; not stated	*Helianthus annuus* L.	Foliar spray: 25 and 45 days after sowing	Improved plant height, leaf area and seed yield; increased proline content; Increased oil yield; decreased Cd, Pb and Cr content in plants; decreased Cr and Pb soil bioavailability	[174]
Cd + Pb	CdSO_4_	1 mg L^−1^ + Pb(NO_3_)_2_; 100 mg L^−1^	Polyvinylpyrrolidone coated NPs; 100 mg L^−1^; HS > 600 nm	*Spinaciae oleracea* L.; *Petroselinum sativum* Hoffm.; *Coriandrum sativum* L.	Hydroponic system (15 days)	Decreased Cd content in shoots and of Pb in roots of cilantro; decreased Cd and Pb content in parley and spinach roots; increased Fe content in parsley and spinach; increased Zn content in all species	[173]
As	NaAsO_2_	2 mg L^−1^	10, 20, 50, 100, 200 mg L^−1^; PS 20–30 nm	*Oryza sativa* L.	Germination and seedling growth in petri dishes	The lowest doses enhanced seedling length and the pigment content, and stimulated the antioxidant response by increasing the activity of SOD and CAT; decreased MDA content and As accumulation and translocation	[146]
	As(V); Na_2_HAsO_4_	25 µM	25 µM (2.0345 mg L^−1^); PS 20 nm	*Glycine max* L.	Hydroponic system (10 days)	Increased root and shoot dry weight; decreased As content in roots and shoots; decreased ROS (H_2_O_2_ and O2^−•^) and MDA in roots and shoots; enhanced the activity of antioxidant enzymes (SOD, G-POX, CAT, APX, GR); upregulated the expression of defense- and detoxification-encoding genes; increased GSH/GSSH, and AsA, proline and glycine betaine contents	[175]
Co	CoCl_2_	300 µM	500 mg L^−1^; PS 20 nm	*Zea mays* L.	Seed priming	Enhanced shoot/root length and biomass; decreased Co bioaccumulation; increased the chlorophyll contents; increased Fv/Fm and gas exchange related parameters, including net CO_2_ assimilation rate; decreased MDA; stimulated the activity of antioxidant enzymes; promoted essential nutrient uptake; restored the ultrastructure of cell organelles, cell guards and stomatal aperture	[176]
Cu	CuSO_4_·5H_2_O	100 mg kg^−1^	50 mg L^−1^; not stated	*Solanum lycopersicum* L.	Foliar spray at 35 days after sowing	Enhanced plant biomass, length, and leaf area; increased chlorophyll index; promoted the gas exchange, including the net CO_2_ assimilation rate; induced an antioxidant response, by increasing antioxidant enzyme activity, proline content; decreased ROS and Cu accumulation	[177]

Under As, nZnO amendment (10–200 mg L^−1^, with higher effects when were used 10–50 mg L^−1^ nZnO) promoted the rice seedlings’ growth, increasing root and shoot biomass, enhanced the chlorophyll levels, and upregulated the activity of CAT and SOD [146]. Furthermore, nZnO reduced the MDA content, as well the levels of As in both roots and shoots, whereas it increased the Zn concentration. In soybean plants, nZnO (2 mg L^−1^) amendment of the nutritive solution containing As (V) reduced several cellular toxicants such as ROS (H_2_O_2_ and O_2_**^−^**^•^), MDA, and oxidized glutathione [175]. On the other hand, in both roots and shoots, several antioxidant pathways were activated, which included the upregulation of the expression of detoxification-encoding genes (*GmSOD*, *GmG-POX*, *GmAPX*, *GmCAT*, *GmGR*, *GmGST*) and the activity of antioxidant enzymes (SOD, G-POX, CAT, APX, GR); the increase of compatible organic solutes (proline and glycine betaine) levels and *GmP5CS* expression; and the stimulation of the ascorbate-glutathione cycle [175].

The single work under cobalt (Co) stress revealed several beneficial effects in maize induced by seed priming with nZnO (500 mg L^−1^) [176]. The pre-treatment with nZnO enhanced maize growth (length and biomass), promoted Zn uptake while reducing Co levels in shoots and roots, and increased chlorophyll contents, which may have contributed to the detected improvement of Fv/Fm. Besides, seed priming reduced the damage induced by Co in guard cells and restored, at some level, the stomatal aperture, as well the chloroplast and thylakoid ultrastructure. These changes may be the reflection of oxidative stress mitigation, as proven by MDA reduction and the superior activity of antioxidant enzymes, and together be responsible for the restoration of gas exchange, including the net CO_2_ assimilation rate [176].

Finally, Cu phytotoxicity was reduced in tomato plants when foliar sprayed with nZnO (50 mg L^−1^) [177]. Treated plants showed lower Cu content, superior length and biomass, higher chlorophyll index, and superior fluorescence of chlorophyll *a*, with the increase of Fv/Fm. However, concerning photosynthesis, nZnO improved the net photosynthetic rate, internal CO_2_ content, stomatal conductance, and transpiration rate, and promoted carbonic anhydrase activity. Seed priming also promoted an antioxidant response with the upregulation of the activities of several enzymes (CAT, APX, and SOD) and the increase of proline, which may have contributed to control ROS production/scavenge (reflect of O_2_**^−^**^•^ and H_2_O_2_ reduction) and reduce the oxidative stress (MDA decrease) [177].

## 4. Protective Effects of ZnO and TiO_2_ against Biotic Stress

Plants are affected by numerous pathogens that are able to induce diseases and diminish plant performance and yield. Crop production is globally affected by pests and phytopathogens such as viruses, bacteria, and fungi, with losses reaching up to 40% of crop local or global production [178,179,180], and thus affecting global food security. NM have been explored as a sustainable alternative to the conventional synthetic agrochemicals, which lack selectivity and sensitivity and are a threat to the environment and human health. This nano-based approach shows desirable properties for agro-application, such as slow and controlled release of active compounds, low cost, efficient drug delivery, multi-site mode of action, ameliorative effects, antimicrobial and/or fungicidal activity, among others [32]. Hence, NM are promising strategies for both plant health monitoring and disease management in smart agriculture. When NM became to be explored for agricultural proposes, these materials were mostly synthesized by conventional methods. Nevertheless, as their potential was revealed, emerged bio-based synthetic methods, where NM were prepared from plants and microbes, as an environmentally-friendly alternative to chemical synthesis with promising results in agricultural fields, such as in crop diseases management [181].

NP induce the generation of ROS, such as hydroxyl, hydroperoxyl, peroxyl, alkoxyl and carbon dioxide radicals, superoxide anions, hydrogen peroxide, and carbonate, and nonradicals, such as ozone, nitric oxide, peroxy nitrite, hypobromous acid, hypochlorite, and organic peroxides [25,182], increasing the level of oxidative stress. Moreover, oxidative stress induces single and double-strand breaks and lesions on nitrogen base and pentose sugar [182], cell damage, injury of cell membrane with leakage of cytoplasmic material, proteins and nuclei acids [183,184]. The accumulation of NP in the membrane of bacteria or fungi induce alterations in cell membrane permeability, leading to disturbances in the proton motive force [182]. Several metal and oxide-NM show direct action against bacteria, fungi, and viruses and even nematodes. Among them are silver (nAg), gold (nAu), cupper (nCu), and nickel (nNi) NP, as well as nZnO (Table 9), nTiO_2_ (Table 10), copper oxide (nCuO), aluminum oxide (Al_2_O_3_), iron oxide (nFe_2_O_3_), and magnesium oxide (nMgO) NP (for review see [32]).

### 4.1. nZnO Potential for Crop Disease Control

nZnO shows antimicrobial activity to plant pathogens, including bacteria and fungi, as well as the nematode *Meloidogyne incognita* [1,185,186,187,188] (Table 9).

In particular, nZnO doped with Fe and Mn showed antibacterial activity against *Pantoea ananatis*, and in the pathosystem *P. anantis*—corn, nZnO doped with Fe, Mn, Cu, or Ni reduced the diseases progression when the NM were foliar sprayed to plants before and after plant inoculation with the bacteria [189]. In the bacterial blight diseases complex of pea, caused by *M. incognita* and *Pseudomonas syringae* pv. *pisi*, nZnO was able to reduce the index diseases and galling population and improve plant growth and pigment content [188]. In tomato, soil amendment with nZnO reduced the diseases incidence induced by *Ralstonia solanacearum* and stimulated plant growth and the antioxidant response (with the decrease of MDA content) and improved soil microbial community [185] (Table 9).

nZnO synthesized from a flower extract presented antibacterial activity against *R. solanacearum* and decreased the bacterial wilt disease in tomato [190], whereas nZnO synthesized from *Citrus medica* peel extracts showed antimicrobial activity against *Streptomyces sannanesis*, *Bacillus subtilis*, *Pseudomonas aeruginosa*, *Salmonella enterica*, *Candida albicans*, and *Aspergillus niger* [191] (Table 9). Biogenic nZnO NPs synthesized from *Trichoderma harzianum*, *Trichoderma reesei*, and co-culture [192], or from *Paenibacillus polymyxa* strain Sx3 [183], showed antibacterial activity against *Xanthomonas oryzae* pv. *oryzae*, responsible for the bacterial leaf blight diseases in rice. Besides, Ogunyemi et al. [183] reported a decrease of bacterial leaf blight diseases in plants foliar sprayed with biogenic nZnO, together with the improvement of plant growth.

CuZn@DEG and ZnO@PEG nanoflowers showed antifungal activity against *Botrytis cinerea* and *Sclerotinia sclerotiorum*, and in lettuce plants inoculated with *S. sclerotiorum*, both NM reduced the disease index and improved the net photosynthesis, photosynthetic quantum yield, and photosynthetic efficiency [193]. The antifungal activity of nZnO was also evaluated against *Fusarium oxysporum* in tomato plants, decreasing the diseases incidence and severity and improving plant growth [194].

Biogenically synthesized nZnO (Table 9) using lemon peel extract showed antifungal activity against *Alternaria citri*, responsible for citrus black rot disease [195], whereas using leaf extract of *Cinnamomum camphora* (L.) Presl, nZnO presented antifungal activity against *Alternaria alternate*, responsible for early blight disease in *Solanum lycopersicum* [184]. In *A. alternate*, nZnO induced an excessive accumulation of MDA and caused the damage of the cell membrane, leading to the leakage of protein and nucleic acid [184]. In addition, ZnO bio-synthesized using *Penicillium chrysogenum* showed antifungal activity against *Fusarium solani*, *Fusarium oxysporum*, *Sclerotium sclerotia*, and *Aspergillus terreus* [196]. An innovative approach in plant defense is the photoactivation of nZnO, and using this approach, it was possible to inactivate *Escherichia coli* B. and *F. oxysporum* in contaminated seeds [197], whereas in strawberry, photoactivated nZnO reduced *B. cinerea* incidences, promoted crop production, and increased fruit shelf-life [198].

**Table 9:** Beneficial application of nZnO on plant diseases management.

NM	Size; Concentrations	Disease Management	Causal Organism	Targeted Plant	Application Method	Outcome	Ref.
nZnO doped with Fe, Mn, Cu or Ni	Not stated; 5, 10 mg L^−1^	White spot	*Pantoea ananatis*	*Zea mays* L.	Leaf spray	Antibacterial activity (nZnO doped with Fe or Mn); reduced disease progression (all).	[189]
nZnO	≤40 nm; 0.01%	Bacterial blight diseases complex	*Meloidogyne incógnita + Pseudomonas syringae* pv. *pisi*	*Pisum sativum* L.	Seed priming; Foliar spray	Antibacterial activity; nematocidal activity Increased plant growth and pigment content; reduced the disease index; reduced galling and nematode populations.	[188]
nZnO	16–31 nm; 500 mg kg^−1^	Bacterial wilt	*Ralstonia solanacearum*	*Solanum lycopersicum* L.	Soil amendment	Enhanced plant growth; decreased MDA content; increased plenylalanine ammonia lyase and POD activity; Increased the richness and diversity of soil microbial communities	[185]
CuZn@DEG and ZnO@PEG	35 nm and 18 nm; 50–1400 µg mL^−1^	Lettuce drop	*Botrytis cinerea; Sclerotinia sclerotiorum*	*Lactuca sativa* L.	Foliar spray	Antifungal activity; reduced the disease index; improved photosynthesis	[193]
nZnO	9–32 nm; 18 µg mL^−1^	Bacterial wilt	*Ralstonia solanacearum*	*Solanum lycopersicum* L.	Soil amendment	Antibacterial activity; stimulated plant growth; reduced bacterial soil population; decreased disease severity	[190]
nZnO	23.44 nm; 100, 1500, 3000 mg L^−1^	Fungal wilt	*Fusarium oxysporum*	*Solanum lycopersicum*	Foliar spray	Antifungal activity; impaired disease development; promoted plant growth	[194]
nZnO	56.1–110.0 nm; 16.0 μg mL^−1^	Bacterial leaf blight	*Xanthomonas oryzae* pv. *oryzae*	*Oryza sativa* L.	Foliar spray	Antibacterial activity; decreased the percentage disease leaf area; improved plant growth	[183]
nZnO	25–450 nm; 7.5 × 10^−3^ M	Gray mold	*Botrytis cinerea*	*Fragaria* × *ananassa*	Foliar and fruit spay	Antifungal activity; reduced disease incidence; improved crop production; increased fruit shelf-life	[198]
nZnO	74.68 nm; 100 μg mL^−1^	Mosaic disease	Tobacco mosaic virus	*Solanum lycopersicum* L.	Foliar spray	Induced systemic acquired resistance (SAR) and reduction of viral accumulation levels and of disease severity; increased plant growth; up-regulated the transcriptional levels of PAL, PR-1, CHS, and POD genes.	[199]

Concerning plant diseases induced by viruses, Abdelkhalek et al. [199] reported the decrease of Tobacco mosaic virus diseases incidence in tomato plants after being sprayed with green-synthesized nZnO. These particles improved plant growth and upregulated tomato-innate defense genes (PAL, PR-1, CHS, and POD) [199].

### 4.2. nTiO_2_ Potential for Crop Diseases Control

The photocatalytic activity of nTiO_2_ contributes to its antifungal and antibacterial activity [1]. For instance, Sar et al. [200] highlighted the antifungal activity of nTiO_2_ (anatase; 3–12 nm; 50, 100, 150, and 200 ppm) against *F. oxysporum* f. sp. radices *lycopersici* and *F. oxysporum* f. sp. *lycopersici*. Application of nTiO_2_ (10–100 nm; 20, 40, 60 and 80 mg L^−1^) in wheat plants reduced the severity of the diseases caused by the fungus *Bipolaris sorokiniana* [201]. Similarly, Hamza et al. [202] demonstrated that these NPs can control *Cercospora beticola* infection in sugar beet (Table 10). Boxi et al. [203] demonstrated that nTiO_2_ at 0.75 and 0.43 mg/plate induces a growth inhibitory effect in two potent phytopathogens: *F. solani*, which causes Fusarium wilt diseases in potato and tomato plants, and *Venturia inaequalis,* which is responsible for apple scab disease. nTiO_2_ foliar application in cucumber (1.6%) and poinsettia and geranium (25 and 75 mM) showed antibacterial action against the pathogenic *P. syringae* pv. *lachrymans* and *Pseudomonas cubensis* and *Xanthomonas hortorum* pv. *pelargonii, Xanthomonas axonopodis* pv. *poinsettiicola* [204,205] (Table 10). The antibacterial activity of TiO_2_ (0.5 mol L^−1^) against the bacteria *Dickeya dadantii,* which causes the stem and root rot diseases in sweet potato, was reported by Hossain et al. [206]. Similarly, nTiO_2_ has a strong antimicrobial activity against nematodes and viruses [32]. Ardakani [207] found nematocidal activity of nTiO_2_ against the root-knot nematode *M. incognita* in tomato plants. nTiO_2_ can also control the pathogenic activities of the virus Turnip mosaic in tobacco plants by limiting the replication of DNA. In faba bean plants, the foliar treatment with nTiO_2_ helped to control the spread of the broad bean stain virus [208]. An insecticide effect of nTiO_2_ was also observed in tomato plants infected with *Bactericera cockerelli* Sulc [209]. The nTiO_2_ treatment induced a high insecticidal effect after 24 h, with a mortality around 93% for the concentrations above 100 ppm.

**Table 10:** Beneficial application of nTiO_2_ on plant disease management.

Size; Concentration	Disease Management	Causal Organism	Targeted Plant	Application Method	Outcome	Ref.
Not stated; 0.5 g L^−1^	Cercospora leaf spot	*Cercospora beticola*	*Beta vulgaris* L.	Foliar spray	Antifungal agent; reduced leaf spots; increased growth and yield	[202]
10–50 nm; 1.6%	Angular leaf spot of cucumber and downy mildew disease	*P. syringae* pv. *lachrymans* and *Pseudomonas cubensis*	*Cucumis sativus* L.	Foliar spray	Antibacterial activity; decreased leaf lesions; improved photosynthesis and chlorophyll levels	[205]
20 nm; 0.02–0.0007%	Root-knot	*Meloidogyne incognita*	*Solanum lycopersicum* L.	Soil	Nematocidal activity Reduced plant weight, and root and stem length	[207]
700 × 900 nm; 150 μM	Broad bean stain disease	Broad bean stain virus (BBSV)	*Vicia faba* L.	Foliar spray	Antiviral activity; reduced stain virus accumulation in leaves; growth improvement	[208]
Not stated; 1000, 100, and 250 ppm	Tomato psyllid	*Bactericera cockerelli*	*Solanum lycopersicum* L.	Foliar spray	Insecticidal effect; increased insect mortality.	[209]

## 5. Conclusions

**Figure 1:**
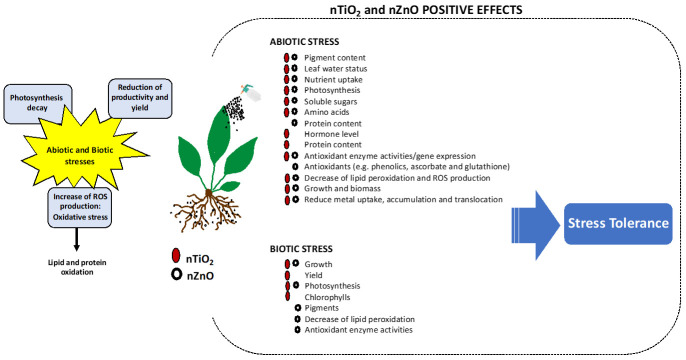
Representative scheme illustrating the positive effects of nZnO and nTiO_2_ application on plant physiology reported for plants grown under abiotic and biotic conditions.

In the last years, nanotechnology has gained much attention in the agro-food system, mostly due to the potential to increase plant performance, enhancing tolerance to biotic and abiotic stresses. In this review, we highlighted the most recent studies on the application of NPs, particularly nZnO and nTiO_2_, in several species exposed to the most common climatic stresses, such as drought and salinity, as well as environmental contaminants, such as heavy metals, and phytopathogens and pests (Figure 1). The beneficial effects of nZnO and nTiO_2_ on plants exposed to these stressors at the molecular, metabolic, and physiological levels are well demonstrated in several works performed under controlled and field conditions. These effects can already depend on several factors such as the type of NP used, method of application, concentration, and the type and extent of stress exposure. In general, these NPs show the potential to improve plant performance and may represent a sustainable strategy to alleviate the negative impacts of (a)biotic stresses in agricultural species (Figure 1).

## Data Availability

This is a review article; however, data used and/or analyzed during the review are available on request from the corresponding author.
